# Internal State of Vesicles Affects Higher Order State of Vesicle Assembly and Interaction

**DOI:** 10.1021/acsomega.4c06037

**Published:** 2024-12-06

**Authors:** Silvia Holler, Federica Casiraghi, Martin Michael Hanczyc

**Affiliations:** † Cellular Computational and Biology Department, CIBIO, Laboratory for Artificial Biology, University of Trento, Via Sommarive 9, Povo 38123, Italy; ‡ Chemical and Biological Engineering, University of New Mexico, Albuquerque, New Mexico 87106, United States

## Abstract

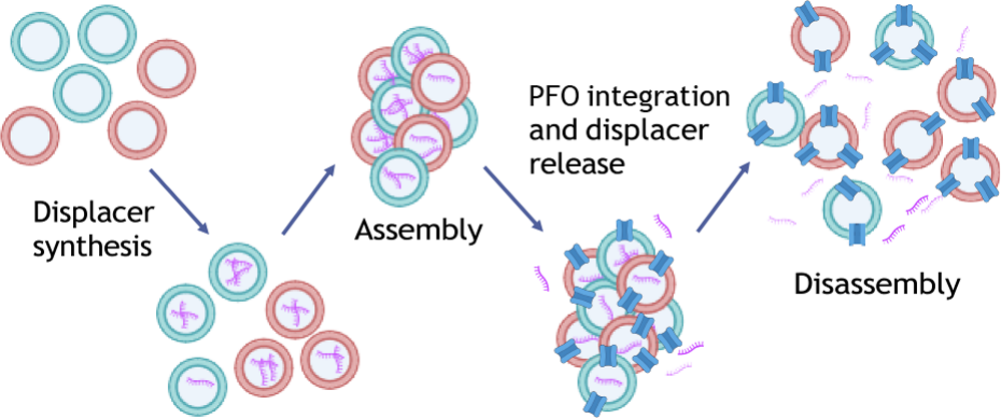

Dynamic soft matter systems composed of functionalized vesicles and liposomes are typically produced and then manipulated through external means, including the addition of exogenous molecules. In biology, natural cells possess greater autonomy, as their internal states are continuously updated, enabling them to effect higher order properties of the system. Therefore, a conceptual and technical gap exists between the natural and artificial systems. We engineered functionalized vesicles to form multicore aggregates capable of self-assembly due to the presence of complementary ssDNA strands. A dynamic process was then triggered through an exogenously triggered on-demand release of an endogenously produced displacer molecule, resulting in multicore aggregate disassembly. This approach explores how internal states of vesicles can affect the external organization, demonstrating a very simple programmable strategy for assembly and then endogenous disassembly. This framework supports the exploration of larger and more complex multicore entities, opening a path toward community behavior and a higher degree of autonomy.

## Introduction

In the quest to understand and manipulate biological systems, synthetic bottom-up models that mimic cellular processes have become invaluable. Among these models, giant unilamellar vesicles (GUVs), stand out due to their ability to encapsulate biomolecules and emulate cell membrane dynamics. Different methods are used to prepare GUVs and include: centrifugation of water-in-oil emulsion droplets,^1^ one-pot synthesis method,^2^ SUV-based hydration,^3^ and electroformation.^4,5^ Vesicles are simplified proxies for living cell membranes, and are now being functionalized for more sophisticated behaviors and applications. Several studies also suggest that as these vesicular systems become more sophisticated they can act as a kind of artificial cell that may not indeed evolve like natural cells, but can mimic some essential features of living cells. In order to emulate natural systems, these vesicles are designed to display behaviors such as performing chemical reactions,^6^ replication,^7^ communication,^8^ responding to stimuli,^9,10^ sensing the external environment,^11^ self-organization,^7^ and targeting specific cells.^12^

In addition to serving as cell membrane proxies, spatially isolated vesicles facilitating chemical reactions can create numerous possibilities for commercial applications.^13,14^ These applications include liposome-mediated drug delivery,^15−18^ the development of multi-component biodevices capable of chemically responding to human body or environmental cues^19,20^ and the communication with real cells.^21^ The organization of GUVs into higher order structures presents a compelling avenue for synthetic biology. These vesicular assemblies can mimic the architecture and functionality of cellular aggregates, offering insights into cellular organization and intercellular communication. Employing techniques such as microfluidics,^22^ self-assembly,^2,23^ and biofunctionalization,^24^ researchers have developed methods to organize GUVs into complex, three-dimensional structures that closely resemble biological tissues.^25^ Upon aggregation, the GUVs might either stay as separate entities^26^ or merge and share lipids.^27^ In much of this research, the vesicles are created and then manipulated through the application of exogenous factors.^26^ In this way, the vesicles show responsiveness and an inherent element of programmability. But unlike natural cells, the vesicles are largely passive and show no autonomy.

The embodiment of autonomy, although more difficult to achieve than programmability, is emerging in bottom-up synthetic biology.^28^ Recently it was shown that activated colloids could engulf giant lipid vesicles as a kind of endocytosis.^29^ Using molecular attachments such as those used in this paper, it has been shown that attached assemblies of active droplets can behave collectively as self-morphic active and interactive systems.^30^ Also using vesicles, self-division was achieved through the encapsulated enzymatic reaction.^31^ In general by changing from passive to active matter, like-life properties of artificial systems are now being demonstrated with aspects of internal organization effecting the overall outcomes.

In this paper, single vesicles were functionalized to participate in more complex aggregates which we call multicore structures. The multicore state was mediated using ligand–receptor pair proxies: through the complementary hybridization of single-strand biotinylated DNA (ssDNA) following a protocol we previously created.^32−34^ In this paper we tested how the assemblies could be affected by the internal state of the vesicles. We show that the aggregates can be modified and disassembled through the release of a RNA displacer, produced on-demand by the vesicles themselves. We present this detailed exploration of expanding the functionality of vesicles, making a contribution to the understanding of multicore aggregate dynamics with an emphasis of internal organization that then affects the external arrangement, as found in living natural cells.

## Results

**Figure 1:**
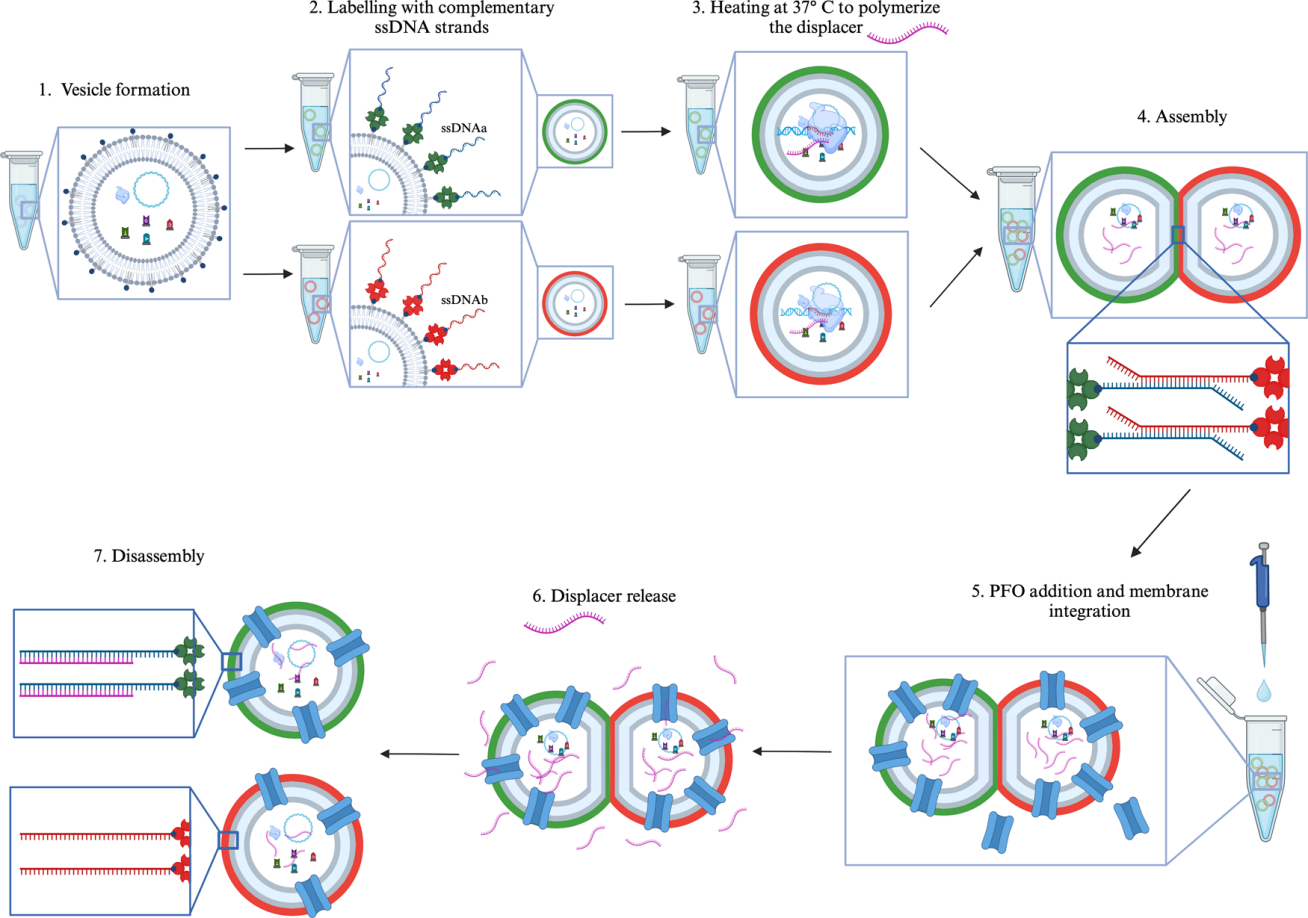
Schematic representation of all the steps to obtain vesicle assembly and endogenously produced RNA-triggered disassembly. Vesicles were formed with transcription machinery encapsulated (step 1). Vesicles were split into two groups, then labeled, using biotinylated lipids and fluorescent streptavidin, with two complementary biotinlylated ssDNA strands (green and red layers surrounding the vesicles, step 2) and heated to favor the displacer production (step 3, pink strand in subsequent steps). Vesicles were mixed and assembled through their complementary ssDNA pairing (step 4). Perfringolysin O (PFO) (blue protein) was integrated into the vesicle membrane (step 5), allowing for the RNA displacer to be released (step 6), and vesicles disassembled due to released displacer disrupting DNA-mediated assembly (step 7).

We established a workflow with the aim to develop an artificial system capable of organizing and reorganizing a higher order multicore structure. Initially vesicles were assembled through complementary DNA pairing as we have shown previously.^34^ In this paper we demonstrate that the disassembly dynamics can be effected through the presence of a toehold, the production and the release of an internally created displacer. All the steps to fulfill this workflow are represented graphically in Figure 1 and described in detail below.

Two different populations of Giant Unilamellar Vesicles (GUVs) were created using the inverted emulsion method with the transcription machinery encapsulated (**step 1**) inside them. Vesicles were tagged with two partially complementary ssDNA strands (**step 2**), which will be referred to as ssDNAa and ssDNAb. Each population was created using a 0.3 mM final concentration of lipids in mineral oil and lipids mixes were enriched with biotinylated lipids to enable the binding of streptavidin and consequently, of the biotinylated ssDNA (13.5 μM final concentration of each ssDNA). ssDNAa and ssDNAb have a complementary binding domain of 17 nucleotides with 33% GC content; moreover, they both have two flanking domains: a 9-nucleotide poly-A spacer (to promote DNA binding, displacement from the vesicle membrane, and avoid steric hindrances), and a 9-nucleotide toehold with a GC content of 50%. The 9-nucleotide toehold is not involved at the early stage in ssDNAa and ssDNAb binding, but will be available at a later step to allow the hybridization of the displacer. To be able to distinguish between the different populations, each was tagged with different fluorescently labeled streptavidins (bound to green Alexa Fluor 488 or to red Alexa Fluor 532). The population tagged with ssDNAa will appear in the reported figures as green and the population tagged with ssDNAb as red. The feasibility of liposome labeling based on ssDNA complementarity through streptavidin binding was reported in our previous papers^32−34^ and was also controlled using fluorescently labeled ssDNA.

In **step 3**, the aim was to produce the required displacer inside the formed vesicles. First, we quantified the displacer production inside vesicles over time, using real-time PCR and SybrGreen II. Different internal and external water phase mixes were used with a fine-tuning of magnesium ion concentration, to optimize the displacer production (Supplementary Figure 1A). We further tested and confirmed that these conditions also support vesicle formation and assembly (in the next step, Supplementary Figure 1B).

After the labeling of the vesicle populations with ssDNAa and ssDNAb and the internal production of the displacer, the vesicles were assembled (**step 4**). The two complementary tagged populations were incubated together for 1 h and 30 min. Supplementary Figure 2B shows a clear representation of the type of assembly obtained after this time period. Supplementary Figure 2A displays an example of vesicle populations (green and blue) not assembled due to labeling with noncomplementary DNA strands as a control. Unassembled vesicles appear round, do not form any kind of agglomerate, and are spread upon the surface of the microscope slide. Instead, GUVs tagged with complementary ssDNAa and ssDNAb (green and red respectively) show a clear signal of population clustering and creation of large agglomerates.

**Figure 2:**
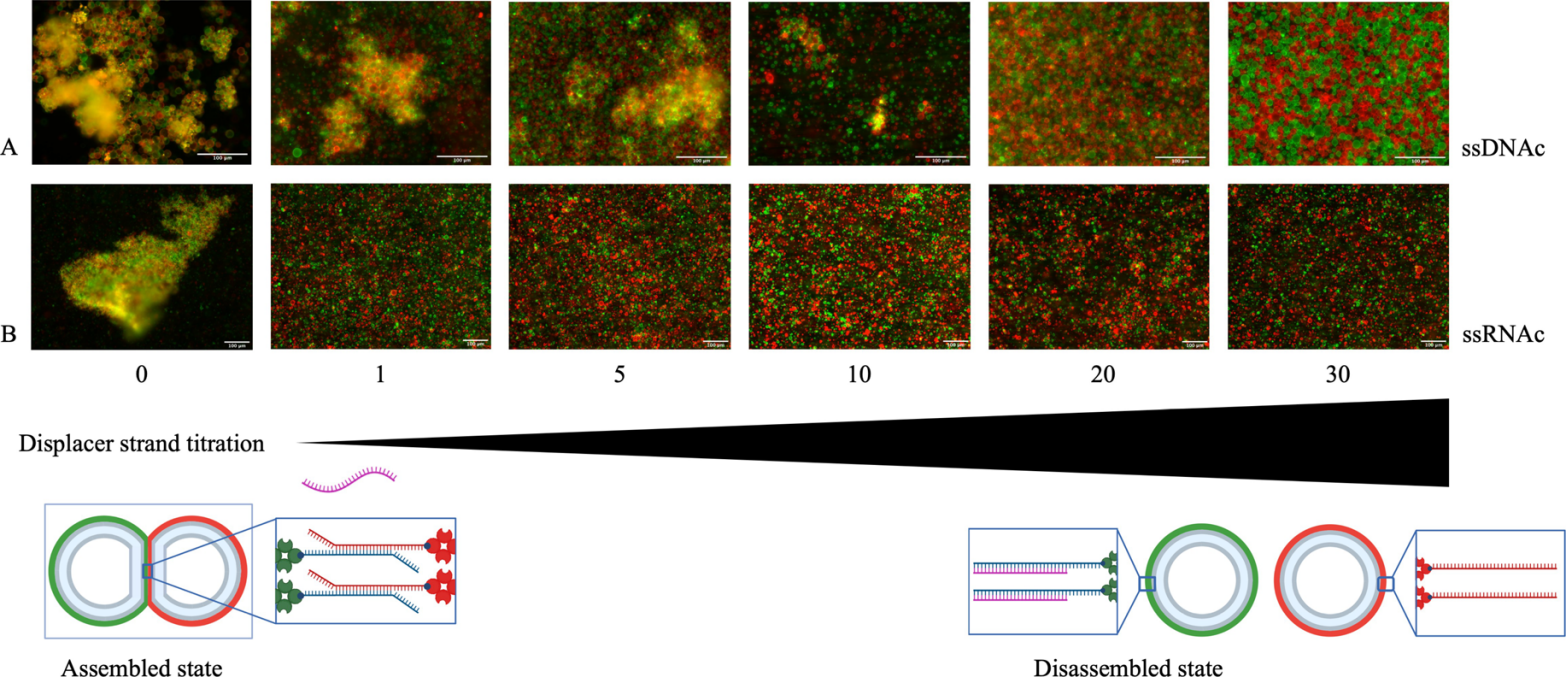
Vesicle disassembly through titration using ssDNAc (A) and ssRNAc (B). Different quantities of ssDNAc and ssRNAc (pink strand) were incubated with previously assembled vesicles for 1 h and 30 min. The fold ratio reported refers to the ratio of ss(DNAorRNA)c to ssDNAa or b quantity (13.5 μM), as reported under each figure column. Scale bar: 100 μm.

At this stage, we analyzed if assembled vesicles could be successfully disassembled using a displacer molecule. The displacer was designed to be fully complementary to the toehold (9 nucleotides) and the central part of ssDNAa involved in ssDNAa-ssDNAb binding (16 nucleotides), with a total length of 25 nucleotides. The displacer was created in two different versions: ssDNAc and ssRNAc of the same sequence. After that vesicles were assembled using ssDNAa and b, various amounts of displacer were titrated, and the system was monitored for disassembly using fluorescence microscopy (see Figure 2). We observed vesicle disassembly using both versions of the displacer, and the RNA displacer disassembled aggregates more efficiently than the DNA equivalent. In the case of DNA, a quantity of nucleic acid 10 times higher than the one used for the assembly was needed to disassemble the vesicles. In the case of RNA, a quantity matching the amount of nucleic acid strands (a and b, 13.5 μM) used for the assembly was instead enough to obtain an efficient disassembly (Figure 2). Hybrid RNA-DNA duplexes have stronger bonding than DNA–DNA duplexes and this can explain the higher displacement efficiency observed in Figure 2.^35^

**Figure 3:**
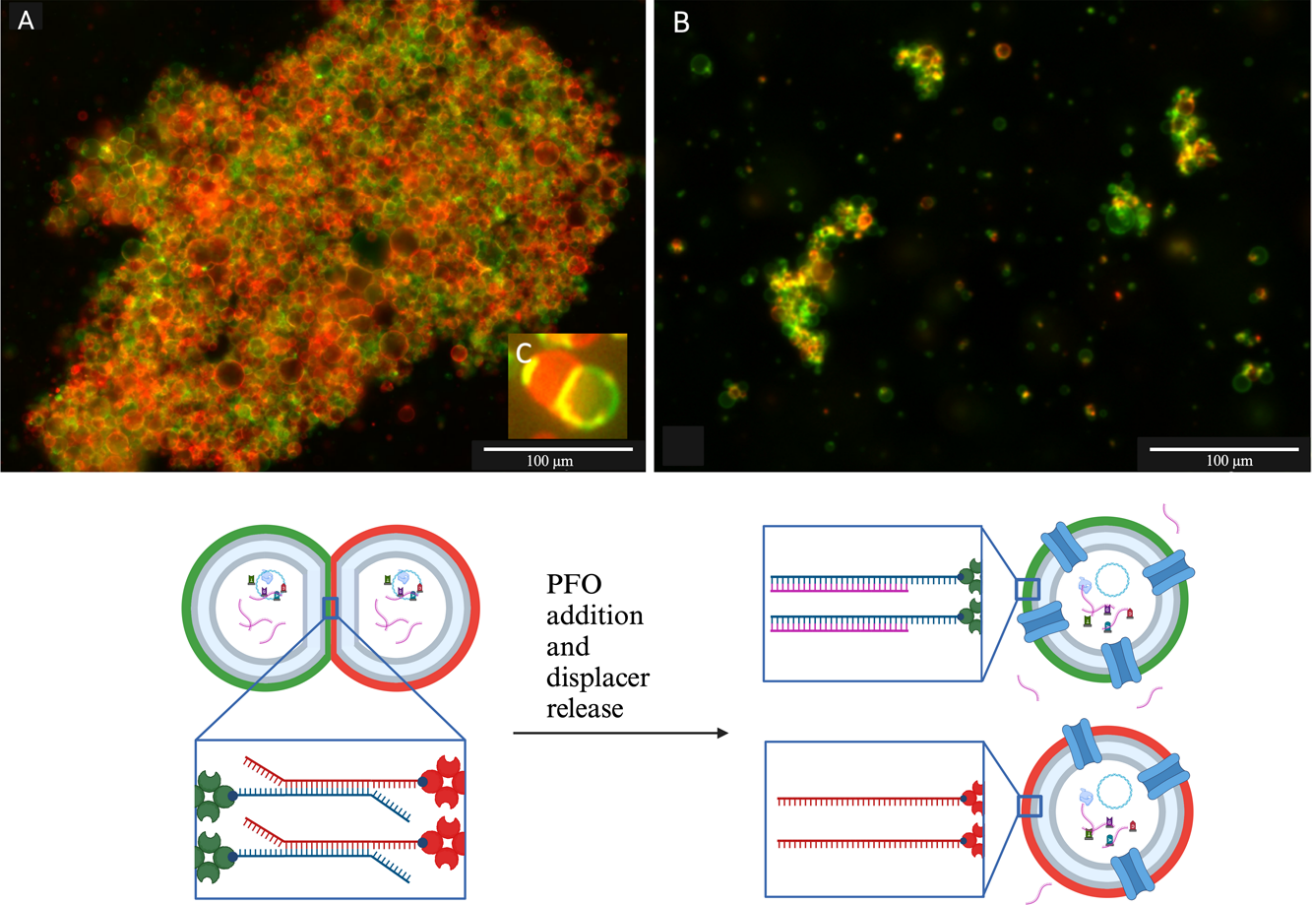
Disassembly of vesicles containing endogenously produced RNA displacer after the addition of the PFO pore. (A) Assembled vesicles after RNA displacer (ssRNAc) synthesis (end of step 4, Figure 1). (B) Vesicle disassembly upon ssRNAc release through PFO inside vesicle membrane (end of step 7, Figure 1). (C) A close-up of the assembled vesicles. (D) Cartoon depicting vesicle assembly and disassembly mediated by PFO integration and ssRNAc release (see also Figure 1). Scale bar: 100 μm.

After having confirmed the higher disassembly efficiency of RNA and having produced assembled vesicles containing internally transcribed ssRNAc, we tested a mechanism for the export of the displacer signal through the vesicle membrane. Short oligomers of nucleic acid and other polyelectrolytes are not able to easily permeate an intact phospholipid membrane. Therefore, we needed to augment our system by the addition of a pore (**step 5**). Perfringolysin O (PFO) was chosen as the pore protein to trigger RNA release. The feasibility of PFO syhthesis inside GUVs and its membrane integration was previously shown by Toparlak et al.^36^ We expressed PFO in *E. coli* and purified it using a His tag and nickel affinity chromatography. The purified pore was then exogenously added to our vesicles and insertion in the membrane and functionality of the protein were afterward tested. Vesicles were created and the PFO integration was assessed through a calcein dequenching assay: calcein release, mediated through PFO integration, was directly related to a fluorescence increase over time. As a positive control for calcein release Triton X-100 was added to purposefully disrupt the vesicles. We empirically determined that ∼3 μM PFO concentration enabled vesicles to maintain their stability but also allowed for macromolecule release. Then vesicles were loaded with ssDNAc or ssRNAc displacers. We tested if PFO addition efficiently released the displacer from the internal lumen of the vesicles and promoted population disassembly (see Supplementary Figure 3). We saw partial disassembly with ssDNAc and total disassembly with ssRNAc. The same experiment with a noncomplementary ssDNAx encapsulated as a control showed no such response. Having confirmed the practicability of all the single steps of our workflow, we proceeded with the test of the whole process to obtain vesicle disassembly through the release of internally synthesized RNA as the displacer (see Figure 3).

Using the previously identified magnesium enriched mix it was possible to generate stable vesicles encapsulating RNA transcription machinery. To produce RNA, GUVs were heated for 3 h at 37 °C, and the quantity of displacer produced was estimated around 0.05 μM. RNA production was estimated after purification using a Nanodrop. Vesicles where then labeled with complementary ssDNA strands a and b, and assembled via incubation for 1 h and 30 min. It is observable that the surface where two vesicles interact is distorted and flattened (see Figure 3C). This flattering is a clear sign of two bound and interacting surfaces forming an adhesion plaque. PFO (3.4 μM) was then added to clusters of assembled vesicles and 3 h later samples were analyzed with a fluorescent microscope. The PFO addition concentration was chosen experimentally in way to match vesicle stability and optimal displacer release. As seen in Figure 3, vesicles are disassembled after the whole process, and the number and the size of assembled clusters are significantly reduced compared to the assembled aggregate that can be visualized in Figure 3a. The quantity of displacer needed for the disassembly was far lower when internally produced (0.05 μM) rather than externally supplied (67.5 μM). Yet this was effective at delivering an effective concentration of displacer proximal to the vesicle adhesion sites. We demonstrated the feasibility and robustness of all the steps of our workflow and also its effectiveness to obtain vesicle disassembly mediated by the release of an internally produced displacer.

## Discussion and Conclusions

Although the reported workflow performs well in the sequence detailed here, other versions of the workflow with steps exchanged did not produce a coherent and functional system. For example, when we tried to polymerize the displacer in situ after vesicle assembly, the incubation at 37 °C decreased the size of the assemblies after a few hours and destroyed the vesicles if incubated overnight. This was due to several factors including vesicle instability over time and at high temperatures and the more obvious factor of DNA melting. Con-sequently we choose to modify the order of our workflow: polymerize the displacer, and then assemble the two populations. Other issues encountered during the workflow also included: balancing vesicle stability with the encapsulation of transcription machinery and the cholesterol concentration necessary for PFO integration and function. GUVs are highly susceptible to breakage if the osmolarity is not balanced. Therefore, polymerization reactions inside of a vesicle can imbalance the osmolarity to the extent that vesicles break and lose contents. In addition it is known that a high amount of cholesterol is necessary for proper PFO membrane integration due to curvature requirements.^37^ However, the inclusion of cholesterol reduces vesicle yield.^38^ Again GUV stability is sensitive to membrane composition and we found that high cholesterol content results in fewer intact GUVs. Having too few vesicles then hampered subsequent steps and manipulations. Therefore, for each step several compromises based on subcomponent compatibility needed to be implemented. In this way we realized that the sequence of steps is critical in producing a functional system with the desired performance.

In conclusion, we previously showed the capacity of these vesicles to interact and assemble in a multicore structures. Here the system has been enhanced to demonstrate the ability to change their higher order state due to an exogenously added pore and internally synthesized effector molecules. This transformation was made possible through the influence of an on-demand produced displacer, showing an on site efficiency far higher than externally supplied oligonucleotides. Thus, this study highlights the potential for an orchestrated multicore organization and responsiveness to external stimuli within aggregates of vesicles, significantly advancing the possibilities within the emerging field of supramolecular chemistry and synthetic biology. These insights have far-reaching implications, moving artificial intelligent materials closer to dynamic living tissues. Possible applications include intelligent artificial tissues that could upon thermal stimulation, given by the normal temperature present in the human body, produce RNA, disassemble, and release drugs or RNA-based therapeutics where needed. Content release will be programmed to release molecules at desired times and locations within the body. This approach could be particularly useful for treatments requiring precise dosage control or localized delivery, such as cancer treatment or hormonal therapy. Furthermore, other disassembly methods could be explored, enabling the on-demand decomposition of a previously synthesized 3D artificial aggregate for tissue regeneration.

## Materials and Methods

The phospholipids POPC (2-oleoyl-1-palmitoyl-*sn*-glycero-3-phosphocholine), DSPE-PEG2000 (1,2-distearoyl-*sn*-glycero-3-phosphoethanol-amine-N-[methoxy(polyethylene glycol)2000]), and DSPE-PEG2000-btn (1,2-distearoyl-*sn*-glycero-3-phosphoethanolamine-N-[biotinyl(polyethylene glycol)2000]), were provided by Avanti Polar Lipids (Alabaster, AL, USA). Cholesterol was purchased in powder form, and solubilized in chloroform to a final concentration of 50 mg/mL. High-quality water (Milli-Q, Millipore, Brussels, Belgium) was used throughout the experiments. Sodium iodide, glucose, sucrose, HEPES, and mineral oil were obtained from Sigma-Aldrich. Streptavidin conjugated to Alexa Fluor was obtainand from Thermo Fischer and all the material for transcription machinery was obtained from Invitrogen. Oligonucleotides were supplied by Explora Biotech (Venice).

### Preparation of the Phospholipid and Aqueous Solutions

Phospolipids (POPC, cholesterol, DSPE-PEG2000 and DSPE-PEG2000-btn) were dissolved in chloroform and mixed in a molar ratio of 50:40:9:1 in a 2 mL glass tube (Fisherbrand, borosilicate amber glass with phenolic cap). Mineral oil (Sigma-Aldrich, Buchs, Switzerland) was added to the glass tube, containing the lipids in chloroform, to obtain a 0.3 mM final concentration of phospholipids. 2% of the total oil volume of dodecane was added to avoid chloroform evaporation. The obtained mix was sonicated (15 min, room temperature) using a Sonorex Digitec DT 156 BH (Bandelin GmbH, Berlin, Germany), and used, within the same day. To perform the inverted emulsion vesicle preparation protocol two types of solutions, with two different densities, were prepared: a hosting solution (HS), containing 25 mM sodium iodide, 0.5 M glucose, and 50 mM HEPES 7.2 pH and an Internal Solution (IS) with a higher density containing 25 mM sodium iodide, 0.5 M sucrose, and 50 mM HEPES 7.2 pH. Depending on the specific steps tested of our workflow, IS composition was slightly varied and balanced osmotically with the HS. For vesicle disassembly triggered by the release of synthetic nucleic acid displacers (Supplementary Figure 3), IS was prepared with 25 mM sodium iodide, 0.5 M sucrose, 150 mM HEPES pH 7.2, and synthetic nucleic acid displacers resuspended in Milli-Q water (100 μM). The quantity of these displacers was 5-times higher (67.5 μM in total of ssDNAx, ssDNAc or ssRNAc) than the quantity of the DNA used to label each population of vesicles (ssDNAa and ssDNAb). For vesicle disassembly mediated by transcribed RNA, ssRNAc was produced through vesicle incubation, prior to the assembly, for 3 h at 37°. Vesicles were prepared encapsulating and containing an IS with the full machinery for transcription: 0.5 M sucrose, 150 mM HEPES pH 7.2, 15 mM MgCl2, 10 mM DTT, 0.2 units RNase inhibitor, 0.5 mM each NTP, 3 units of T7 RNA Polymerase and 5 nM of purified plasmid.

### Giant Unilamellar Vesicles (GUVs) Preparation

Vesicles were prepared through inverted emulsion method. For the creation of a water-in-oil droplet emulsion, IS (20 μL) and mineral oil enriched with 0.3 mM phospholipids (400 μL) were mixed in an eppendorf tube and emulsified by mechanical scratching on an eppendorf holder. The obtained emulsion was then added to an eppendorf tube where 200 μL of HS were previously placed. The obtained system was centrifuged 1 min at 16,000 rcf (room temperature) and vesicles formed and pelleted on the bottom of the eppendorf. The oil phase remaining on the top of the eppendorf was removed by aspiration using a vacuum pump and subsequent 3 washing steps in HS of the obtained pellet performed. These steps consisted of HS addition, centrifugation (2 min, 5000 rcf, room temperature), supernatant removal using a vacuum pump, and HS addition.

### Surface Functionalization

For surface functionalization of the biotinylated phospholipid enriched (DSPE-PEG2000-btn) membranes, streptavidin, streptavidin Alexa Fluor 488, 532, and 350 conjugates were used. Oligonucleotides were dissolved to a final concentration of 100 μM in Milli-Q water.

The ssDNA or ssRNA sequences were the following:

- ssDNAa 5′ CGAAGTTCCAATAGTAATCTGTCGTTAAAAAAAAA biotin 3′
- ssDNAb 5′ GCTTCAGACAACGACAGATTACTATTAAAAAAAAA biotin 3′
- ssRNAc 5′ ACGACAGAUUACUAUUGGAACUUCG biotin 3′ (and 5′ fluorescein in the case of externally synthesized displacer used for -disassembly experiment in Supplementary Figure 3)
- ssDNAc 5′ ACGACAGATTACTATTGGAACTTCG biotin 3′ (and 5′ fluorescein in the case of externally synthesized displacer used for disassembly experiment in Supplementary Figure 3)
- ssDNAx 5′ TGGAGGGCTCTTTCT 3′ with a biotin at 5′

ssDNAa and ssDNAb were created, starting from the sequences used by Hadorn et al. 2012, to be partially complementary having 15 nucleotides (with 33% GC content) of perfect complementarity, and two flanking domains: a 10-nucleotide poly-A spacer (to promote DNA binding, displacement, and avoid steric hindrances), and a 10-nucleotide toehold domain (with a GC content of 50%). This 10 nucleotide domain is not involved in DNAa and DNAb binding, and is available as a toehold. ssRNAc and ssDNAc, the displacer strands, are 35 nucleotides in length and are designed to be fully complementary to ssDNAa. The labeling solution (streptavidin and DNA) was prepared with a specific volume ratio, streptavidin:ssDNA 2.7:1, as reported in Hadorn et al., 2012. To obtain vesicle labeling equal volumes of GUVs and labeling solution were mixed (100 and 100 μL) and a solution with 13.5 μM final ssDNA concentration was obtained. Eppendorf were afterward incubated for 1 h in the dark. Differently fluorescently tagged vesicle populations were obtained. Vesicles were washed twice using a centrifugation step (2 min, 5000 rcf, RT), removing the old supernatant, and replacing it with fresh HS. The functionalized vesicles were used within a day.

### Protein Purification and Quantification

PFO was produced and purified using a PFO expressing plasmid, with His-tag, and BL21 (DE3) pLys *E. coli*. PFO expression was induced with 1 mM IPTG at 37° C overnight. BL21 cells were pelleted, lysed, and centrifuged (17000 g for 30 min at 4°). The obtained solution was filtered using 0.2 μm syringe filters and loaded onto a Ni-NTA chromatography column previously equilibrated with Buffer A (50 mM Tris-HCl pH 8.0, 750 mM NaCl, 25 mM Imidazole). After adding the sample containing PFO on the column, an extensive wash with Buffer A was performed, and the protein was then eluted using Buffer B containing 300 mM Imidazole. To check for the PFO expression, all the fractions were denatured by heating them at 95° for 5 min using Cracking buffer 6X (Tris HCl pH 7.5 300 mM, Glycerol 50%, SDS 0.5%, β-mercaptoethanol 5%, Bromophenol blue 0.3 mg/mL, DTT 0.093 g/mL), loaded on SDS-PAGE precast gel, and run. Fractions were analyzed by staining with Comassie Brilliant Blue. Fractions having proteins with size correspondence to PFO were dialyzed in SnakeSkin Dialysis Tubing (3.5K MWCO, 35 mm) and a buffer containing 50 mM Tris HCl pH 8.0, 750 mM NaCl, 5 mM DTT and 30% Glycerol. Protein concentration was determined through the Bradford method and absorbance reading using a plate reader (Varioskan LUX Multimode Microplate Reader). The obtained solution was aliquoted and stored at −80°.

### Assembly and Disassembly Processes and Microscopy Visualization

Vesicle populations tagged with complementary or non complementary DNA were incubated for 2 h at room temperature in an eppendorf tube. 3.4 μM PFO was afterward added to clusters of assembled vesicles and 3.5 h later samples were analyzed. Aggregates were transferred to a sealed microscopy observation chamber by gentle aspiration without resuspension. The microscope chamber was composed as follows: a silanized microscope slide, a silicon spacer, 20 μL of vesicle solution, and a microscope coverslip 24 × 60 mm (MenzelGlas̈er, Braunschweig, Germany). The samples were evaluated using an upright light and fluorescence microscope Zeiss Axio Imager M2 with a X-CITE 120Q light source.

## Supporting Information Available

Test for conditions that support displacer (ssRNAc) production; vesicle assembly test; and vesicle disassembly triggered by PFO integration and displacer release (PDF)
