# Supplementary material for: Internal State of Vesicles Affects Higher Order State of Vesicle Assembly and Interaction
Source: ACS Omega. 2024 Dec 6;9(50):49316–22. doi: 10.1021/acsomega.4c06037 (PMC11656350; doi:10.1021/acsomega.4c06037)
Supplement: Supplementary file 1 — ao4c06037_si_002.pdf [file ao4c06037_si_002.pdf]

Internal state of vesicles affects higher order state of vesicle assembly and interaction

Silvia Holler,[†,¶] Federica Casiraghi,[†,¶] and Martin Michael Hanczyc[*,†,‡]

†*Cellular Computational and Biology Department, CIBIO, Laboratory for Artificial Biology, University of Trento, Via Sommarive 9, Povo, 38123, Italy.*

‡*Chemical and Biological Engineering, University of New Mexico, Albuquerque, NM, 87106, USA.*

¶*Contributed equally to this work*

E-mail: martin.hanczyc@unitn.it

Phone: +0461 283438

Supporting Information Available

This document contains:

Supplementary figures 1-3.

Supplementary Figure 1

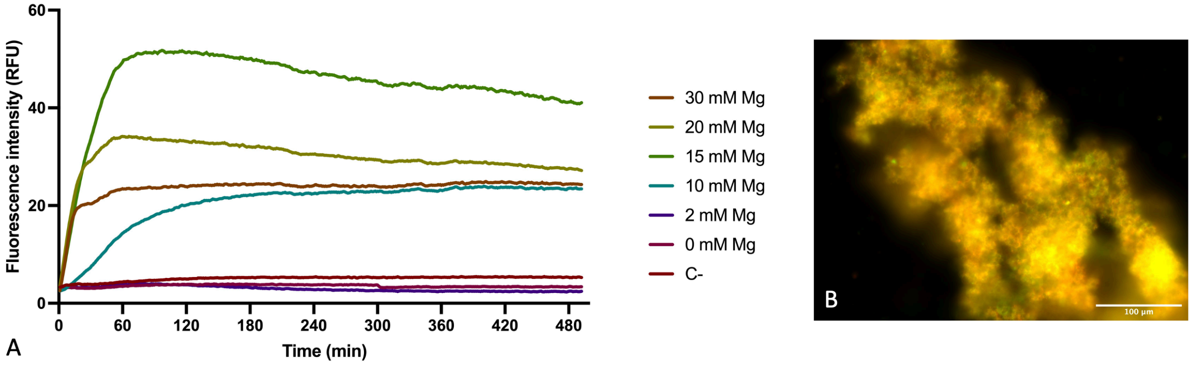

Supplementary Figure 1. Test for conditions that support displacer (ssRNAc) production. A) ssRNAc production over time with varying magnesium chloride concentrations. RNA production was monitored using SybrGreen II and a PCR cycler. B) Assurance test of vesicle assembly while using 15 mM magnesium in the internal and external solutions. Scale bar: 100 μm.

Supplementary Figure 2

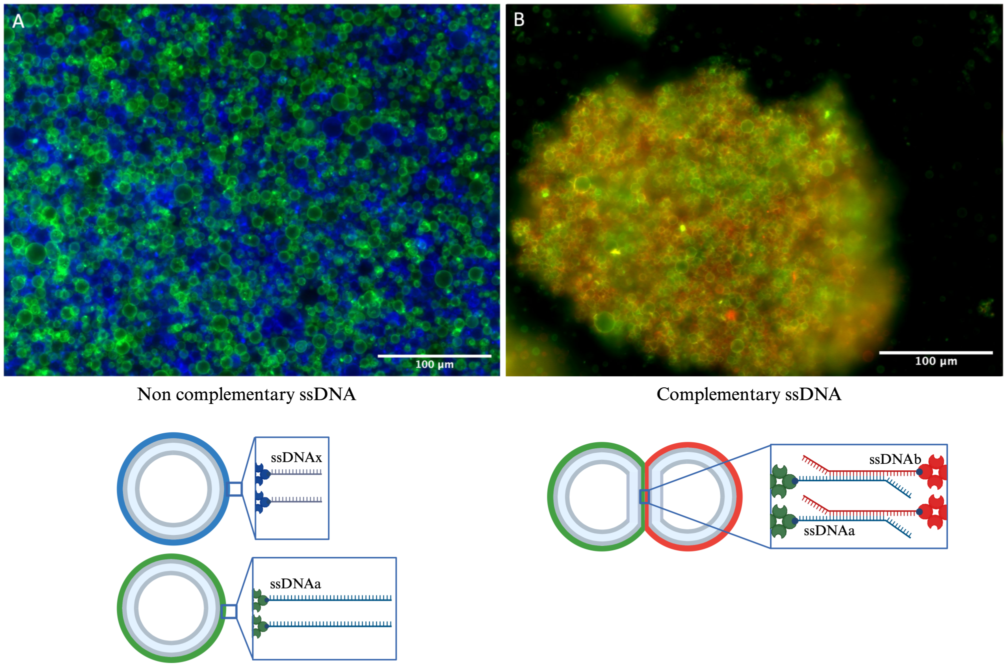

Supplementary Figure 2. Vesicle assembly test. A) vesicles not assembled due to non-complementary DNA (blue and green vesicle populations, bound to ssDNAx and ssDNAa respectively through Alexa Fluor 350 and 488 streptavidin); B) assembled vesicles due to complementary ssDNAa and ssDNAb (green and red vesicle populations, Alexa Fluor 488 and 532 streptavidin). Scale bar: 100 μm.

Supplementary Figure 3

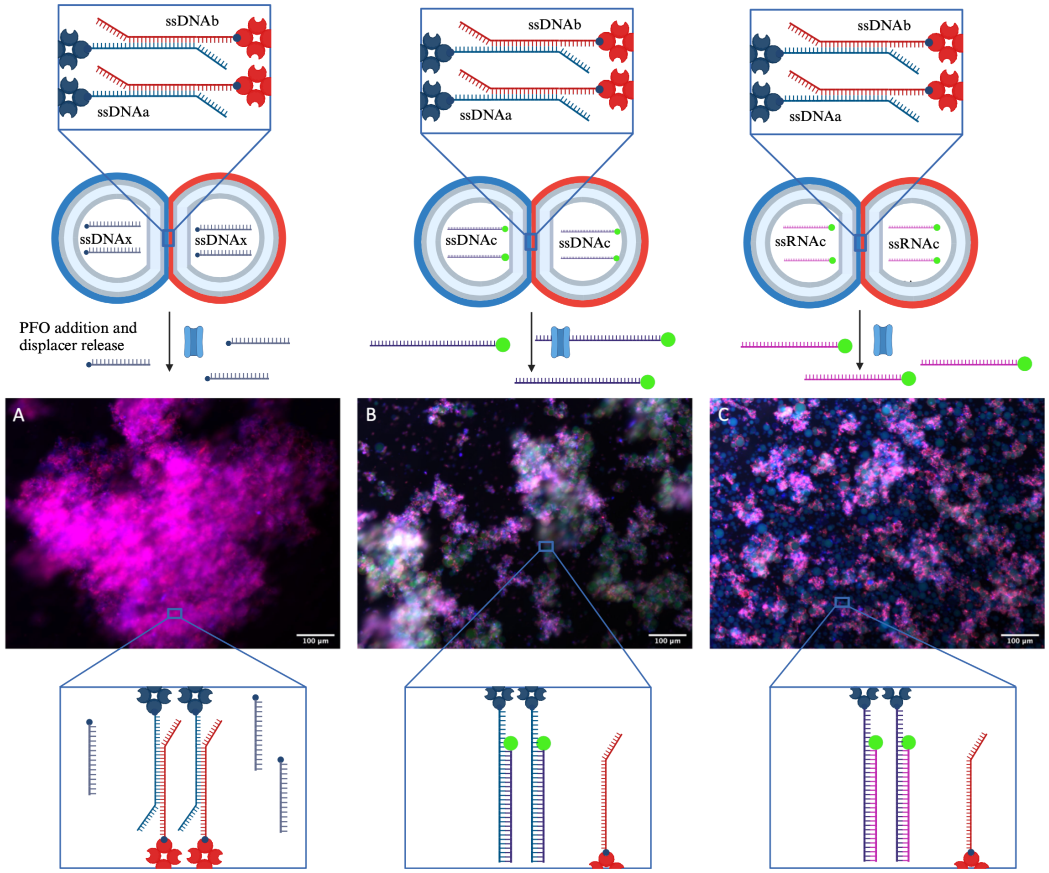

Supplementary Figure 3. Vesicle disassembly triggered by PFO integration and displacer release. Two vesicle populations, tagged with streptavidin Alexa Fluor 532 and 350, were assembled using complementary ssDNAa and ssDNAb (blue and red populations). Vesicles were also loaded with externally produced RNA and DNA displacers (ssRNAc and ssDNAc, tagged with fluorescein to visualize them) while they are released from vesicles after PFO addition and membrane insertion. A) Negative control: assembled vesicles after release of unrelated ssDNAx. B) Disassembly triggered by the release of DNA displacer ssDNAc. C) Disassembly triggered by the release of RNA displacer ssRNAc. Scale bar: 100 μm.
